# Cardiac involvement in Wilson disease: pathophysiology, clinical spectrum, and multimodality imaging assessment

**DOI:** 10.3389/fcvm.2026.1884519

**Published:** 2026-08-14

**Authors:** Daiping Hua, Lanting Sun, Shang Xiang, Ye Yuan, Chunsheng Xu, Qiaoyu Xuan, Wenming Yang, Han Wang

**Affiliations:** 1 Department of Neurology, The First Affiliated Hospital of Anhui University of Chinese Medicine, Hefei, China; 2 Center of Imaging, The First Affiliated Hospital of Anhui University of Chinese Medicine, Hefei, China; 3 Key Laboratory of Xin'an Medicine, Ministry of Education, Anhui University of Chinese Medicine, Hefei, China

**Keywords:** cardiac involvement, cardiac magnetic resonance, copper metabolism, myocardial fibrosis, Wilson disease

## Abstract

Cardiac involvement in Wilson disease (WD), a rare autosomal recessive disorder of copper metabolism, has been historically underrecognized but is increasingly shown to be clinically significant. This review synthesizes the current understanding of the pathophysiology, clinical presentations, and the important but still evolving role of multimodality imaging in assessing cardiac disease in WD. Myocardial copper deposition directly and via secondary oxidative stress leads to cardiomyocyte injury, fibrosis, and electrical instability. The clinical spectrum ranges from subclinical abnormalities to overt cardiomyopathy, life-threatening arrhythmias, and sudden cardiac death. Electrocardiography often reveals conduction abnormalities and repolarization changes. Echocardiography, including advanced techniques like speckle-tracking, can detect subtle systolic and diastolic dysfunction, often before a decline in ejection fraction. Cardiovascular magnetic resonance (CMR) offers unique advantages for non-invasive tissue characterization and has shown promise in detecting myocardial abnormalities in WD, with studies consistently demonstrating increased extracellular volume, native T1/T2 values, and late gadolinium enhancement—particularly at the right ventricular insertion points and interventricular septum—indicating diffuse fibrosis and inflammation; however, its role in routine clinical practice remains to be standardized. Recent CMR studies also suggest concomitant coronary microvascular dysfunction. This review underscores the importance of systematic cardiac evaluation in WD patients, integrating clinical assessment with multimodality imaging to enable early detection, risk stratification, and timely intervention.

## 1 Introduction

Cardiac involvement in Wilson disease (WD) represents a critical yet historically underrecognized dimension of this systemic disorder, necessitating urgent and focused investigation. WD is an autosomal recessive inherited disorder caused by mutations in the *ATP7B* gene (1, 2), which impair biliary copper excretion (3, 4) and lead to progressive copper accumulation in the liver and subsequently in extrahepatic organs, including the heart (5, 6).

Historically, clinical attention and research initiatives have primarily concentrated on the hepatic and neurological consequences of WD, with diagnostic criteria and therapeutic frameworks developed accordingly (1, 7–9). As a result, the cardiotoxic potential associated with chronic copper accumulation was traditionally perceived as a minor or infrequent complication, leading to its systematic underrecognition in both clinical practice and research. Nevertheless, an expanding body of evidence now unequivocally establishes the heart as a significant and well-recognized target organ (10–12). Cardiac involvement in WD encompasses a range of manifestations, including arrhythmias, cardiomyopathy, autonomic dysfunction, and fatal cardiac events (11, 13–15), which collectively contribute substantially to patient morbidity and mortality (16, 17). Despite evidence indicating a spectrum of complications from subclinical dysfunction to sudden cardiac death (SCD) (14, 18), these cardiac issues are frequently overlooked in routine clinical management.

With advancements in pathological studies and the revolutionary progress in modern cardiovascular imaging—particularly the application of cardiac magnetic resonance (CMR) (19–21)—cardiac involvement in WD has transitioned from sporadic case reports to a well-defined clinical entity (10, 12, 22–25). Accumulating evidence unequivocally identifies the myocardium as a target of copper toxicity (26, 27), thereby establishing cardiac involvement as an integral component of the WD phenotype. This review aims to systematically elucidate the pathophysiological mechanisms underlying cardiac involvement in WD, comprehensively delineate its broad clinical spectrum, which ranges from subclinical abnormalities to life-threatening events, and critically appraise the current and potential roles of multimodal imaging—including electrocardiogram (ECG), echocardiography, and CMR—in early detection, precise characterization, and risk stratification, while acknowledging the current lack of standardized protocols for routine CMR use. The ultimate objective is to provide an evidence-based framework to optimize integrated care and improve long-term outcomes for patients with WD.

## 2 Pathophysiology of cardiac involvement in WD

### 2.1 Mechanism of copper metabolic disorder and cardiac pathological injury in WD

WD is characterized by a disruption in copper homeostasis due to mutations in the *ATP7B* gene, which impair the function of the *ATP7B* protein (28, 29). This protein is essential for the incorporation of copper into ceruloplasmin and the facilitation of biliary copper excretion (2, 30). Under normal physiological conditions, copper is absorbed in the gastrointestinal tract, binds to albumin, and is transported to the liver (31, 32). In the liver, *ATP7B* plays a crucial role in integrating copper into ceruloplasmin for systemic distribution or excretion into bile (2, 33, 34). In individuals with WD, the impaired excretion of copper into bile results in its progressive accumulation within the liver. Once the hepatic storage capacity is surpassed, excess copper is released into the bloodstream and subsequently deposited in extrahepatic tissues, including the brain, kidneys, and heart (5–7, 29). Although the liver and brain are primarily affected, recent evidence highlights the significance of cardiac copper accumulation as a notable factor contributing to mortality in WD (14, 16, 35).

The mechanisms through which copper accumulation induces cardiac damage are complex, with both direct toxic effects and secondary pathways playing pivotal roles. Post-mortem studies have documented myocardial copper concentrations up to 100 times higher than normal (23), and direct accumulation within cardiomyocytes is hypothesized to disrupt cellular machinery and promote apoptosis (36, 37). Circulating free copper catalyzes the production of reactive oxygen species (ROS) via the Fenton and Haber-Weiss reactions, damaging cellular lipids, proteins, and DNA (36, 38–40). This oxidative stress specifically targets cardiomyocytes and the extracellular matrix, leading to structural and functional alterations (41, 42). A systematic review of 21 studies has underscored oxidative stress and mitochondrial dysfunction as principal pathological mechanisms in WD (43). Mitochondria are particularly vulnerable: copper accumulation within the matrix impairs the electron transport chain by inhibiting cytochrome c oxidase (Complex IV), resulting in reduced ATP production essential for myocardial contraction (44–46). Concurrently, oxidative stress synergizes with energy depletion to induce endoplasmic reticulum stress, shifting the unfolded protein response toward apoptosis and exacerbating cardiomyocyte loss (47, 48). Moreover, mitochondrial copper overload disrupts calcium homeostasis and triggers permeability transition pore opening, leading to cardiomyocyte necrosis and further worsening myocardial dysfunction (49, 50).

In addition to these mechanisms, copper overload in cardiomyocytes can induce cuproptosis, a recently identified form of copper-dependent regulated cell death (36, 39, 51). This process plays a critical role in WD pathogenesis (52). Mechanistically, excessive intracellular copper causes toxic aggregation of lipoylated mitochondrial enzymes, such as DLAT, and the loss of Fe-S cluster proteins, leading to lethal proteotoxic stress (53, 54). In WD models, markers of cuproptosis (FDX1, DLAT, lipoic acid synthase) are upregulated, and knockdown of cuproptosis-related genes (e.g., Lox, App, Gpc1, Gls) mitigates cell death *in vitro* (52). Although direct evidence in cardiac tissue is lacking, their conserved function implies a possible role in WD-related myocardial injury.

Copper overload in WD also severely disrupts autophagy. Initially, ROS activates autophagy as a protective measure against damage, but prolonged excessive autophagy can promote cell death (55, 56). This transition is driven by autophagy-dependent ferroptosis (57, 58). Ferroptosis has been implicated in various cardiovascular diseases (59, 60). Systemic iron homeostasis disturbances, common in WD (61, 62), may increase the labile iron pool, sensitizing cardiomyocytes to ferroptotic damage. Autophagy can selectively target anti-ferroptotic proteins (ferritin and GPX4), compromising antioxidant defenses (57, 63, 64). Copper itself can also trigger ferroptosis (6, 32). Consequently, in the WD heart, copper toxicity may activate a network of cuproptosis, dysregulated autophagy, and ferroptosis that collectively exacerbate myocardial injury.

Dysregulation of the autonomic nervous system (ANS), affecting both sympathetic and parasympathetic branches, contributes to arrhythmias, abnormal heart rate variability (HRV), and orthostatic hypotension. These effects may occur independently or synergistically with direct myocardial damage (15, 65–67). For instance, research in critical illness has demonstrated that baroreflex dysfunction can lead to increased expression of G-protein-coupled receptor kinase 2 (GRK2) in cardiomyocytes, driven by nicotinamide adenine dinucleotide phosphate oxidase subunit-2-mediated oxidative stress (68). Given that GRK2 is a key negative regulator of cardiac β-adrenergic receptors, its upregulation impairs contractile function and promotes arrhythmias (68, 69). This pathway represents a plausible mechanism linking the chronic autonomic dysregulation and oxidative stress observed in WD to its clinical cardiac manifestations.

### 2.2 Histopathological features of cardiac involvement in WD

The histopathological changes of cardiac involvement in WD are characterized by a spectrum of copper-mediated myocardial injury. These changes, ranging from early subcellular damage to advanced structural remodeling, are primarily confirmed by post-mortem examinations (11, 23, 70, 71), endomyocardial biopsy findings (10), and pathological analyses of WD animal models (72). The most fundamental histopathological hallmark is abnormal copper accumulation in the myocardium. As early as 1959, Bottiger and Mollerberg (70) first documented increased myocardial copper content in a 17-year-old male with WD at autopsy, and postulated this direct metal deposition as a probable cause of the concurrent cardiac hypertrophy. Kaduk et al. (23) (1980) described myocardial lesions in WD, pointing out that mitochondrial changes are a consequence of myocardial copper deposition. Furthermore, post-mortem studies using atomic absorption spectrophotometry confirmed that the myocardial copper concentration in WD patients can be as high as 100 times that of healthy individuals. Factor et al. (71) documented non-specific but definitive myocardial alterations, including interstitial and replacement fibrosis, small-vessel sclerosis, and focal inflammation, with cardiac hypertrophy present in over half of the specimens.

Crucially, advanced non-invasive imaging, particularly CMR, has allowed the *in vivo* characterization of this pathological spectrum in living patients. Additionally, a prospective CMR study identified left ventricular (LV) clefts—invaginations penetrating more than 50% of the wall thickness of the adjoining compact myocardium in diastole—in 20% of WD patients (12/61) compared to 5% of controls (3/61) (73). Although these clefts were incidental findings in asymptomatic patients, their high prevalence in WD suggests a potential developmental or degenerative impact of copper on myocardial architecture.

Collectively, cardiac involvement associated with WD is pathologically characterized by copper-induced myocardial injury, which includes abnormal myocardial copper accumulation and subsequent alterations such as mitochondrial damage, fibrosis, and cardiac hypertrophy. These pathological features were initially identified through post-mortem examinations, endomyocardial biopsies, and analyses of animal models. Furthermore, advanced non-invasive imaging techniques, particularly CMR, have facilitated the *in vivo* characterization of these pathological changes, even in asymptomatic WD patients. CMR has been instrumental in detecting specific indicators, such as LV clefts.

## 3 Clinical spectrum of cardiac involvement in WD

This section systematically examines the primary clinical features of cardiac involvement in WD, detailing its epidemiological distribution and distinguishing between symptomatic and asymptomatic phenotypes. It further explores the clinical characteristics and prognostic implications of various key clinical manifestations, thereby offering a foundation for clinical identification and risk stratification.

### 3.1 Epidemiology of cardiac manifestations in WD

Historically regarded as rare, cardiac involvement in WD is now acknowledged as a prevalent and clinically significant component of the disease spectrum, impacting a substantial proportion of patients across all age groups. The prevalence of detectable cardiac abnormalities in WD varies significantly depending on the sensitivity of the screening tools used, illustrating a broad disease continuum. Studies utilizing clinical symptoms, standard ECG, or administrative coding report abnormalities in approximately one-third of patients, primarily reflecting overt or clinically recognized disease (11, 35, 74). Early prospective research by Kuan et al. identified ECG abnormalities in 34% (18/53) of WD patients, including findings such as ventricular hypertrophy, ST-T changes, and various arrhythmias (11). Retrospective cohort studies support this range. For instance, Wang et al. reported that 29.4% (37/126) of adult WD patients exhibited either overt cardiac symptoms (e.g., chest tightness, palpitations, dizziness) or asymptomatic ECG abnormalities, with the latter comprising the majority of cases (74). This prevalence aligns with results from other ECG-based studies conducted in both adult and pediatric cohorts (13, 75, 76). Moreover, a comprehensive, population-based database analysis in the United States demonstrated that patients with WD exhibit significantly increased risks of arrhythmias, heart failure (HF), autonomic dysfunction, and cardiac arrest relative to the general population (35).

In stark contrast, systematic evaluation using sensitive CMR imaging reveals a high prevalence of myocardial injury in WD, including in asymptomatic individuals. In a seminal prospective CMR study, Quick et al. demonstrated that 95% (58/61) of WD patients exhibited pathological findings (12). Another extensive CMR study conducted by Salatzki et al. reported that 41% (31/76) of patients displayed late gadolinium enhancement (LGE) (77). Furthermore, in a rigorously selected cohort of asymptomatic, well-treated patients assessed with advanced multiparametric CMR, Johansson et al. found a 71% (12/17) prevalence of LGE (25, 78). Collectively, these findings suggest that while clinically apparent disease manifests in approximately one-third of patients, objective evidence of myocardial involvement is detectable in the vast majority when appropriate imaging technology is employed. This epidemiological evidence underscores the critical importance of proactive cardiac assessment in all WD patients, utilizing tools of appropriate sensitivity to capture the full scope of cardiac risk. Furthermore, an analysis of the California Healthcare Cost and Utilization Project database demonstrated that patients with WD exhibit a threefold increased risk of developing cirrhosis. Notably, even after statistically adjusting for the presence of cirrhosis—a recognized cause of cardiac dysfunction through cirrhotic cardiomyopathy—the risks of HF remained significantly elevated (16).

### 3.2 Clinical phenotypes: symptomatic vs. asymptomatic cardiac involvement in WD

Asymptomatic cardiac involvement is a common phenotype in WD patients (12, 74, 77), defined as the presence of abnormal cardiac structure, function, or electrical activity detected by imaging or electrophysiological examinations without any cardiovascular-related complaints (such as palpitations, chest tightness, or dyspnea). It accounts for more than half of WD patients, especially in the early stage of the disease or those with relatively stable condition under standardized treatment (12, 79). Although such patients have no immediate clinical symptoms, subclinical injuries can progress continuously, serving as important potential risk factors for future HF, arrhythmias, or even SCD (14, 16, 80).

The primary complaints among symptomatic patients exhibit considerable diversity, predominantly encompassing palpitations (primarily associated with arrhythmias), exertional dyspnea, and reduced exercise tolerance (largely linked to ventricular dysfunction resulting from cardiomyopathy), as well as syncope or amaurosis fugax [chiefly attributable to ventricular arrhythmias (VA), severe atrioventricular block, or orthostatic hypotension] (74, 77, 79). In more severe cases, patients may present with lower extremity edema and other symptoms indicative of HF (77, 79, 80). Prospective studies have provided quantifiable insights into this burden; for example, in a systematic CMR cohort, Quick et al. reported that 12% of patients with WD experienced dyspnea at rest or during exertion, 24% experienced dizziness, 15% had a history of syncope, and 13% reported palpitations (77). Retrospective analyses support these findings. A retrospective cohort study involving 126 adult WD patients revealed that 29.4% (37/126) exhibited cardiac manifestations, with 23.0% (29/126) being asymptomatic and 7.1% (9/126) presenting with symptomatic disease (74). Among the symptomatic patients, a range of non-specific symptoms were reported, including intermittent chest tightness (3/9), dizziness (3/9), palpitations (2/9), and atypical chest pain (1/9) (74).

The severity of symptoms is closely associated with the overall severity of WD, particularly demonstrating a significant correlation with the Unified WD Rating Scale (UWDRS) score (12, 22, 77, 79). Elevated UWDRS scores are linked to an increased incidence and severity of cardiac symptoms, as well as a notable rise in the frequency of supraventricular and ventricular premature beats (12, 22). This relationship highlights that symptomatic cardiac involvement frequently indicates a more advanced or severe systemic disease state.

### 3.3 Core clinical manifestation patterns of cardiac involvement in WD

In WD, both symptomatic and subclinical cardiac injuries ultimately manifest in four primary and frequently overlapping clinical patterns: arrhythmias, cardiomyopathy, autonomic dysfunction, and SCD, with the latter representing the most severe outcome (11).

#### 3.3.1 Arrhythmias

Arrhythmias are among the most prevalent cardiac manifestations in WD, presenting a spectrum from asymptomatic subclinical abnormalities to life-threatening malignant arrhythmias (74, 81, 82). Compared with the general population, WD patients have a threefold higher risk of arrhythmias and a 29% higher risk of developing atrial fibrillation (16, 35). Cohort studies report a prevalence ranging from approximately 30% to over 40% (74–76). A significant proportion of WD patients with arrhythmias remain asymptomatic, with abnormalities often detected incidentally through routine ECG or 24-h Holter monitoring (76, 83, 84). Clinically, WD-related arrhythmias encompass a comprehensive range of atrial, ventricular, and conduction abnormalities (11). Atrial arrhythmias and conduction disturbances are relatively common, including ectopic activities such as atrial premature beats and atrial fibrillation, as well as indicators of abnormal atrial depolarization, such as inverted P-waves, bifid P-waves, and prolonged P-wave dispersion (11, 74, 75, 83). Arat et al. have notably demonstrated that asymptomatic patients with WD exhibit significantly increased P-wave dispersion compared to healthy controls, suggesting this may indicate an early stage of cardiac involvement (83). This finding is supported by the work of Karhan et al., who also identified increased P-wave dispersion and prolonged QT intervals in asymptomatic pediatric WD patients, emphasizing that these changes may predict an elevated risk of arrhythmias (84).

VAs, while sometimes considered benign in the absence of structural heart disease, are of significant clinical concern due to their potential prognostic implications. These arrhythmias primarily include premature ventricular contractions (PVCs), non-sustained ventricular tachycardia (VT), and, in more severe cases, polymorphic VT or ventricular fibrillation (VF) (65, 81, 85). Additionally, conduction blocks constitute another critical category, encompassing Mobitz type I atrioventricular block, second- or third-degree atrioventricular block, and central atrial block (11, 74, 82). For example, Kuan identified various conduction blocks in patients with WD (11), and a case report documented second-degree Mobitz type I atrioventricular block as the cause of dizziness in a young female patient with WD (82). Notably, a recent case study by Bantidos et al. described a 40-year-old male patient with genetically confirmed WD who exhibited a spontaneous type 3 Brugada ECG pattern, which converted to type 1 following a flecainide challenge, despite having no prior history of arrhythmia or adverse cardiac events during follow-up (86).

Special populations exhibiting distinct arrhythmia characteristics encompass pediatric patients and those with WD who present with neurological involvement. Pediatric patients with WD demonstrate a notable incidence of arrhythmias, reported at 43.1% in one study, with sinus tachycardia and sinus bradycardia being the most prevalent, followed by bifid P-waves, ST-segment alterations, and occasional PVCs (75). Importantly, VA may serve as the initial clinical manifestation in young children with WD, often leading to misdiagnosis if metabolic or genetic causes are not considered (81). In patients with neurological manifestations of WD (WD-neuro^+^), research by Ozturk et al. revealed significantly prolonged QT and corrected QT (QTc) intervals compared to hepatic manifestation only (WD-neuro^−^) patients and healthy controls, suggesting an elevated risk of ventricular repolarization disorders and associated arrhythmias (87). Furthermore, a study by Ertaş et al. on pediatric WD patients indicated that, despite normal ventricular and autonomic function, this group exhibited significantly increased QT dispersion (QT-d) and T peak-to-T end interval (Tp-e), implying a heightened risk of VA (85).

The prognostic risk of WD-related arrhythmias is intricately linked to the severity of the disease, the specific type of arrhythmia, and the occurrence of acute exacerbations. Firstly, severe arrhythmias, such as VF and high-grade atrioventricular block, are direct risk factors for SCD (11, 35). Secondly, even arrhythmias considered benign, such as PVCs, are relevant to prognosis. Quick et al. highlighted that PVCs in WD patients are associated with heightened risks of ventricular dysfunction, sudden death, and all-cause mortality (65). Furthermore, there is a positive correlation between disease severity and the risk of arrhythmias; the severity of WD, as measured by the UWDRS, significantly correlates with the frequency of PVCs (22). Patients with a history of acute exacerbations of WD exhibit elevated troponin levels and may be more susceptible to severe arrhythmias (22, 65). Consequently, early identification and management of arrhythmias are essential for improving the prognosis of patients with WD.

#### 3.3.2 Cardiomyopathy

Cardiomyopathy constitutes the primary structural manifestation of cardiac involvement in WD, resulting from direct copper-induced myocardial toxicity and subsequent fibrotic remodeling. This condition is predominantly characterized by LV remodeling, hypertrophy, and dysfunction (11, 14, 70, 71). A systematic review encompassing 32 studies identified cardiomyopathy as the most prevalent structural abnormality among WD patients (14). Subclinical myocardial involvement is highly prevalent in WD and often precedes overt cardiac symptoms, emphasizing the value of early detection via sensitive imaging modalities. A prospective CMR study of 61 WD patients revealed that despite normal LV ejection fraction (LVEF) and absence of cardiac symptoms (12), the majority exhibited signs of myocardial involvement, such as elevated T1, T2, and extracellular volume (ECV) values. These findings indicate early myocardial edema, inflammation, and fibrosis, which have the potential to progress to overt cardiomyopathy if not addressed. Notably, even after standardized copper chelation therapy or liver transplantation, WD patients may still experience severe cardiomyopathy or cardiac arrest (88, 89), underscoring the irreversibility of chronic myocardial injury and the imperative for long-term monitoring.

In WD, overt cardiomyopathy typically manifests as either dilated or hypertrophic cardiomyopathy, accompanied by HF symptoms such as exertional dyspnea, fatigue, and lower extremity edema. Pediatric patients with WD also demonstrate early structural cardiac changes, with 16% of asymptomatic children exhibiting concentric LV remodeling (84). Moreover, LV clefts, identified in 20% of WD patients, constitute a distinctive structural abnormality that may be associated with chronic copper toxicity (73); however, their clinical significance warrants further investigation. Although rare, dramatic presentations such as Takotsubo (stress) cardiomyopathy induced by fulminant hepatic failure have been reported, highlighting the heart's susceptibility during systemic WD crises (88).

Moreover, in the assessment of cardiomyopathy associated with WD, it is essential to consider the potential presence of concurrent, genetically distinct cardiac disorders. A recent case report has documented the co-occurrence of WD alongside a pathogenic variant in the *FLNC* gene, known to be linked with inherited dilated cardiomyopathy (90). This observation highlights the importance of employing comprehensive genetic testing methodologies, such as whole-genome sequencing, to elucidate the etiology of cardiomyopathy in WD patients, particularly in instances characterized by atypical or severe clinical manifestations.

#### 3.3.3 Autonomic dysfunction

Autonomic dysfunction is a prevalent yet frequently overlooked cardiac manifestation in WD, with a notable incidence of subclinical involvement (15, 91). A systematic review of 14 studies involving 297 patients with WD indicated that the incidence of ANS abnormalities ranges from approximately 8% to 79.2%, with significant variability attributable to differences in assessment methods and study populations (15). A large population-based study corroborated these findings, demonstrating that autonomic dysfunction occurs in 4.9% of WD patients, with an OR of 3.7 compared to the general population, underscoring its clinical significance (35). Importantly, the majority of autonomic dysfunction cases associated with WD are asymptomatic or oligosymptomatic, often going unreported by patients and detectable only through specialized autonomic function tests (AFTs) (15). Furthermore, Matarazzo documented abnormal circadian rhythms of temperature, pulse, and blood pressure in WD patients exhibiting psychiatric manifestations, proposing hypothalamic dysfunction as a potential pathogenic mechanism (92).

The clinical spectrum of WD-related autonomic dysfunction is extensive, involving cardiovascular, sudomotor, visceral, and small fiber components. Among these, cardiovascular manifestations are the most thoroughly characterized, including symptoms such as postural dizziness, syncope, orthostatic hypotension, and abnormal HRV (67, 93–95). For example, Bhattacharya et al. reported that 29% of 14 patients with WD exhibited abnormal autonomic reflexes, evidenced by reduced HRV during controlled breathing, an abnormal Valsalva ratio, and the absence of blood pressure overshoot during the fourth phase of the Valsalva maneuver (93). Oligosymptomatic orthostatic hypotension is also prevalent, with 19% of 43 WD patients demonstrating asymptomatic orthostatic hypotension in a prospective study (11). Severe autonomic dysfunction may occasionally present as the initial symptom (95). Sudomotor and visceral symptoms, including abnormal sweating, xerostomia, xerophthalmia, abdominal cramps, bladder and bowel dysfunction, and erectile dysfunction, are frequently observed; however, these symptoms are often underestimated (15, 67, 93). From an electrophysiological perspective, autonomic dysfunction is characterized by prolonged sympathetic skin response (SSR) latency, abnormal RR interval variation (RRIV), and diminished baroreflex sensitivity (66, 67, 91). While some studies suggest predominant sympathetic involvement (66), others report more pronounced parasympathetic impairment (94). Small fiber dysfunction has also been documented (96, 97). Additionally, a pilot study by Kouvelas et al. on young WD patients revealed abnormal autonomic regulation of blood pressure (98).

In specific populations characterized by unique autonomic dysfunction profiles, WD-neuro^+^ patients and those undergoing prolonged anti-copper treatment are noteworthy. WD-neuro^+^ patients have a markedly higher prevalence of autonomic dysfunction than WD-neuro^−^ patients (15, 67, 91), with one study reporting rates of 37.5% versus 5.6% (67). Longitudinal data indicate that these abnormalities—namely, elevated nocturnal heart rate, reduced expiration-inspiration ratio, and diminished baroreflex sensitivity—persist even with optimal maintenance therapy (91). Matarazzo observed that WD patients presenting initially with psychiatric symptoms frequently experience concurrent circadian rhythm disturbances in vital signs, which may aid in differential diagnosis (92). In pediatric patients with WD, Ertaş et al. observed that, despite normal ventricular and autonomic function, the low-frequency to high-frequency ratio, an indicator of sympathovagal balance, was significantly elevated, suggesting latent autonomic dysregulation (85). Case reports suggest that early anti-copper therapy may reverse autonomic dysfunction, with normalization of SSR and symptoms reported after 6 months (95) and 36 months (99) of treatment, respectively. However, baroreflex sensitivity may continue to decline despite overall functional stability (91).

The prognosis of WD-related autonomic dysfunction is intricately linked to the severity of the disease, the extent of neurological involvement, and the timing of treatment initiation. While the majority of autonomic dysfunction cases are subclinical, severe manifestations, such as refractory orthostatic hypotension and syncope, can substantially impair quality of life (67, 95, 97). Epidemiological data suggest a higher prevalence of autonomic dysfunction in WD among Caucasians, adults, and females, indicating a need for heightened surveillance in these subgroups (35). Notably, the association between the severity of autonomic dysfunction and the overall clinical severity of WD is limited (94), implying that autonomic evaluations should be conducted independently of assessments for hepatic or neurological symptoms. This underscores the necessity for comprehensive clinical evaluations and objective AFTs, such as SSR, the Valsalva maneuver, HRV analysis, and skin wrinkling tests, to prevent misdiagnosis (100).

#### 3.3.4 SCD

SCD represents the most severe and life-threatening complication associated with cardiac involvement in WD (80, 89). It is primarily induced by malignant arrhythmias or progressive cardiomyopathy (11, 71, 80) and frequently occurs without preceding overt cardiac symptoms (14), thereby presenting substantial challenges for early detection and intervention.

Epidemiological evidence confirms the non-negligible incidence of SCD in WD patients. A national population-based study encompassing 1,030 individuals with WD revealed that 10 experienced cardiac arrest, equating to a prevalence of approximately 0.97%. Furthermore, WD patients exhibited an OR of 2.4 for cardiac arrest compared to the general population, highlighting a markedly increased risk of life-threatening cardiac events (35). Similarly, Kuan's prospective study of 53 consecutive WD patients identified two cases of cardiac death (11). Importantly, population-based data indicate that HF in WD is more prevalent among Caucasians and females, suggesting that these subgroups warrant heightened surveillance for life-threatening cardiac involvement (35). The clinical characteristics of WD-related SCD are marked by an insidious onset and a variety of triggering factors. Chevalier's comprehensive literature review documented three instances of SCD in WD patients, all occurring abruptly without prodromal symptoms and with no definitive non-cardiac causes of death identified (14), further implying that sudden death in WD is likely attributable to cardiac etiologies.

Furthermore, SCD can manifest in specific clinical contexts: Bobbio et al. documented a case of cardiac arrest in a 38-year-old male WD patient one year post-liver transplantation, which was ascribed to persistent myocardial copper accumulation and subsequent myocardial fibrosis. This case underscores that the risk of SCD persists even following successful liver transplantation (89). Adar et al. reported a case of Takotsubo cardiomyopathy, also known as broken heart syndrome, complicating fulminant hepatic failure in a 16-year-old female patient with WD. This condition represents a rare yet potentially life-threatening cardiac involvement that may progress to SCD if not promptly addressed (88).

## 4 Multimodality imaging in the assessment of cardiac involvement in WD

This section examines the fundamental importance of multimodal imaging technologies in assessing cardiac involvement in WD, elucidating the clinical positioning, identifiable pathological indicators, and applicable scenarios for each examination based on the latest clinical studies. Notably, CMR is increasingly recognized as a valuable non-invasive tool for evaluating cardiac structure and function (101). Consequently, this section will address three key aspects: first-line screening with ECG, comprehensive structural and functional evaluation with echocardiography, and advanced tissue characterization with CMR. It will elaborate on the respective roles and application characteristics of these modalities in assessing cardiac involvement in WD, with a particular emphasis on the evolving utility of CMR in pathological quantification and the detection of subclinical injury.

### 4.1 Primary screening: ECG

The ECG serves as a fundamental, non-invasive screening instrument for assessing cardiac involvement in WD. It is primarily employed to rapidly detect abnormal myocardial electrical activity and to offer preliminary insights that guide subsequent, more detailed examinations. The ECG is appropriate for routine screening across all age groups of WD patients (13, 74, 75), and is particularly advantageous for implementation in primary healthcare settings and large-scale population screenings. As a first-line screening modality, the ECG can initially identify various conduction abnormalities in patients with WD. However, the specific nature of these abnormalities necessitates further clarification through arrhythmia-specific evaluations.

ECG abnormalities in patients with WD exhibit considerable diversity and are characterized by specific age-related and phenotypic variations. In pediatric patients with WD, ECG abnormalities demonstrate distinct specificity, with T-wave abnormalities (35.2%) and sinus tachycardia (23.5%) being the most prevalent, followed by sinus bradycardia, ST-segment changes, and occasional ventricular premature beats (75). Notably, the QT-d and Tp-e intervals in pediatric patients are significantly elevated compared to healthy controls, indicating an increased potential risk for VA (85). In adult WD patients, there is a higher incidence of QRS widening, atrial premature beats, ventricular premature beats, and atrioventricular block (74, 84). Among these, P-wave dispersion, an indicator of atrial conduction disturbance, is significantly prolonged even in asymptomatic WD patients (83). Furthermore, QTc interval prolongation is more prominent in WD-neuro^+^ (87), which can be used as a key screening indicator for cardiac involvement in WD-neuro^+^ patients, providing a basis for subsequent further evaluations.

The ECG exhibits inherently low sensitivity and specificity in evaluating cardiac involvement associated with WD. However, a normal ECG does not preclude the existence of structural myocardial abnormalities, as it primarily reflects disturbances in myocardial electrical activity rather than structural alterations such as ventricular wall thickening, LV enlargement, or myocardial fibrosis. Indeed, some WD patients with normal ECGs may exhibit abnormal strain on echocardiography or fibrosis on CMR (24, 84). Consequently, the ECG should not be utilized as the sole diagnostic tool for assessing cardiac involvement in WD. Instead, it should be integrated with echocardiography, CMR, or other imaging modalities to facilitate a comprehensive evaluation.

### 4.2 Comprehensive structural & functional evaluation: echocardiography

Echocardiography, as a non-invasive, real-time imaging modality, is fundamental for the comprehensive structural and functional assessment of cardiac involvement in WD. It addresses the limitations of ECG, which only reflects electrical activity, by providing direct visualization of myocardial structure, valvular function, and cardiac hemodynamics (76, 102, 103). This technique is crucial for confirming cardiac involvement, assessing disease severity, and guiding therapeutic decisions (18, 84, 104), and is commonly employed in clinical practice as a secondary evaluation tool following ECG screening. The primary advantage of echocardiography lies in its capacity to integrate structural observation with functional measurement, facilitating precise evaluation of both macroscopic structural abnormalities and subtle functional impairments associated with WD-related cardiac injury. However, conventional echocardiography may appear normal in the early stages of the disease, whereas advanced echocardiographic techniques have expanded the ability to detect subclinical myocardial involvement (18, 103–105).

The structural assessment of cardiac involvement related to WD via echocardiography primarily concentrates on abnormalities in the myocardium, valves, and cardiac chambers. Myocardial hypertrophy is among the most prevalent structural anomalies, frequently manifesting as LV hypertrophy (LVH) (76, 102). In patients with WD, echocardiographic indicators of myocardial hypertrophy are typically characterized by increased thickness of the interventricular septum and the LV posterior wall (76), with a minority of patients exhibiting eccentric hypertrophy accompanied by LV dilation (84). In pediatric patients with WD, echocardiography can also detect early structural abnormalities, such as concentric LV remodeling. A study involving 43 asymptomatic pediatric WD patients revealed that 16% (7/42, with one patient excluded from the remodeling analysis) exhibited concentric remodeling of the LV. Compared to healthy controls, the patient cohort demonstrated increased relative LV wall thickness, although no significant differences were observed in LVEF or diastolic function (84). Furthermore, myocardial echocardiographic abnormalities typically present as diffuse or focal increases in myocardial echo intensity, uneven distribution, and reduced echo uniformity (103). Ultrasonic backscatter analysis, a noninvasive ultrasound tissue characterization technique derived from videodensitometry, facilitates the early detection of myocardial changes by quantifying myocardial texture through the analysis of gray-level distribution in echocardiographic images. A study specifically compared 18 cardiologically asymptomatic patients with WD to 15 age-matched healthy controls. The findings indicated that the WD group exhibited a significantly higher corrected mean gray level at end-systole (cMGLs) in the LV posterior wall compared to the control group (30.9 ± 2.6 vs. 22.2 ± 2.7, *p* = 0.033). Additionally, the WD group demonstrated a significantly lower cyclic variation index (CVI) in the interventricular septum (−22 ± 4.4% vs. 43.4 ± 12.9%, *p* < 0.001), although no significant difference was observed in the posterior wall CVI between the two groups (103). Furthermore, valvular involvement is also possible in WD patients, with mild valvular regurgitation being a common occurrence (65).

Functional evaluation constitutes a critical aspect of echocardiography in patients with WD, encompassing both systolic and diastolic function assessments. These evaluations can identify subclinical cardiac dysfunction more effectively than ECGs. In the assessment of systolic function, the LVEF serves as the primary indicator. Most WD patients exhibiting early cardiac involvement maintain a normal LVEF (18, 87, 104); however, those with significant myocardial damage or advanced disease may exhibit reduced LVEF (84), indicative of LV systolic dysfunction. Techniques such as tissue Doppler imaging (TDI) and speckle-tracking echocardiography (STE) demonstrate greater sensitivity in detecting early systolic dysfunction. A study involving 43 asymptomatic pediatric WD patients revealed that conventional echocardiographic parameters (LVEF, shortening fraction, and diastolic function) were comparable to those of control subjects. Nonetheless, TDI identified reduced mitral lateral and septal systolic annular velocities (*p* = 0.02 and *p* = 0.04, respectively) and increased isovolumetric contraction time (*p* = 0.04), suggesting subclinical systolic dysfunction (84). These TDI abnormalities are regarded as early indicators of myocardial damage, potentially preceding alterations in conventional echocardiographic parameters (18, 105, 106). Moreover, STE is capable of detecting subtle abnormalities in myocardial strain or strain rate in WD patients who exhibit normal LVEF, thereby offering critical insights for the early diagnosis of subclinical cardiac involvement (18, 104, 105). In particular, a study concentrating on strain and strain rate echocardiography in pediatric WD patients demonstrated that STE can identify reduced end-systolic longitudinal strain in specific myocardial segments, such as the basal lateral and basal septal segments, as well as abnormal radial strain-related parameters, including decreased end-systolic rotation, even when LVEF and other conventional indicators of systolic function remain within normal ranges (105).

Echocardiographic findings in patients with WD exhibit distinct variations across specific populations. In pediatric patients with WD, echocardiographic abnormalities are predominantly mild and subclinical, and STE has been shown to facilitate early detection in this population (18, 106). Among patients with WD-neuro^+^, echocardiography frequently reveals more pronounced diastolic dysfunction compared to those without neurological involvement (84). In asymptomatic WD patients with normal ECGs, echocardiography can uncover subclinical structural or functional abnormalities, such as changes in myocardial echogenicity, which are critical for early intervention and improved prognosis (84, 103). In summary, echocardiography functions as a comprehensive screening and monitoring modality, with advanced techniques enhancing the early identification of subclinical cardiac involvement.

Despite its significant clinical utility, echocardiography presents certain limitations in assessing cardiac involvement associated with WD. Echocardiography has limited sensitivity for detecting early or diffuse myocardial fibrosis, and its imaging quality may be compromised in patients with thoracic deformities, pulmonary emphysema, or obesity (22, 65).

### 4.3 Advanced tissue characterization with CMR: current evidence and clinical considerations

CMR offers superior spatial resolution, tissue characterization capability, and reproducibility compared with echocardiography (12, 101, 107). Unlike echocardiography, which is constrained by imaging quality and operator dependence, CMR facilitates a multi-parametric, quantitative evaluation of myocardial fibrosis, edema, copper-induced tissue damage, and subtle ventricular dysfunction (22, 77, 108), even at the early subclinical stage of cardiac involvement (12, 22, 108). CMR has been extensively applied to characterize cardiac phenotypes in WD (12, 22, 24, 25, 73, 77, 79, 108–110).

#### 4.3.1 Structural assessment

The structural assessment of cardiac involvement related to WD using CMR primarily concentrates on ventricular geometry, myocardial thickness, and specific structural abnormalities. This method yields consistent and reliable results that are unaffected by variations in body habitus or thoracic deformities.

In terms of LV structure, the majority of WD patients exhibit an increased LV mass compared to controls, although very few meet the diagnostic criteria for LVH (12, 24, 77). A modest but significant increase in interventricular septal thickness has also been reported (77).

Notably, LV clefts, a specific structural abnormality, have been increasingly identified in patients with WD through CMR imaging. In a prospective controlled trial, 20% of WD patients (12/61) exhibited LV clefts, characterized as myocardial invaginations penetrating more than 50% of the adjacent myocardial thickness during diastole, predominantly located in the septal segments, inferior, lateral, and anterior walls (73). In contrast, this abnormality was observed in only 5% of healthy controls (3/61), indicating that the heightened incidence of LV clefts may represent a potential pathological marker of WD. However, the clinical significance and long-term prognostic implications of these findings require further investigation (73, 108).

Regarding the right ventricular (RV) structure, CMR assessments indicate that WD patients infrequently exhibit significant RV dilation, although abnormalities at the RV insertion point (RVIP) are relatively common (12, 22, 24, 77, 79, 108).

#### 4.3.2 Systolic and diastolic function evaluation

CMR offers a more precise and comprehensive evaluation of cardiac systolic and diastolic function in patients with WD, particularly in identifying subtle ventricular dysfunction that conventional echocardiography may overlook.

Regarding global systolic function, the majority of WD patients, including those with neurological involvement, exhibit normal LVEF and RVEF (77, 79, 108, 110). Only a minority present with mild to moderate reductions in LVEF, which is more prevalent among WD-neuro^+^ patients (77, 109).

Nevertheless, studies have reported inconsistent findings concerning LVEF. Most large-cohort studies have found no significant difference in LVEF between WD patients and controls. For instance, in a study involving 61 WD patients, only 5 individuals had an LVEF below 57% (12), and in another cohort of 76 WD patients, only 4 WD-neuro^+^ patients exhibited slightly decreased LVEF (77). Conversely, independent small-cohort CMR studies (25, 78), comprising 17 WD patients, indicated that LVEF was significantly lower in WD patients compared to healthy controls. The observed discrepancy in LVEF findings is more likely attributable to differences in sample size and patient selection, as smaller cohorts may have inadvertently included patients with more advanced disease or suboptimal treatment, rather than reflecting true biological heterogeneity.

A study conducted by the Quick team, which analyzed the same cohort of 61 patients across two sequential reports (12, 24), documented a reduction in RVEF. This included the identification of two patients with an RVEF below 40%, alongside a significant impairment in RV systolic function as measured by RVEF, RV fractional area change, and tricuspid annular plane systolic excursion (12, 24). In contrast, a larger study involving 76 WD patients by Salatzki et al. (77) found no significant difference in RVEF between the overall WD group and healthy controls. However, when patients were stratified by clinical phenotype, those with WD-neuro^+^ exhibited significantly lower RVEF (77). This finding suggests that RV dysfunction may be more pronounced in WD-neuro^+^ patients, potentially indicating more severe systemic copper accumulation.

#### 4.3.3 Strain analysis for subclinical dysfunction

CMR-based strain analysis, incorporating feature tracking and strain-encoded imaging techniques, can detect subtle ventricular dysfunction in WD patients who exhibit normal EF (22, 24, 25, 77–79, 109, 110). Nevertheless, the results of strain parameters have shown considerable variability across studies.

Using fast-SENC (strain-encoded imaging), Salatzki et al. (109) found no significant difference in RV global circumferential strain (RV-GCS) between the overall WD cohort and healthy controls; however, WD-neuro^+^ patients demonstrated significant reductions in both LV-GCS and RV-GCS. In a subsequent study expanding the cohort to 76 patients, the same team confirmed that LV-GCS remained significantly diminished, while LV-global longitudinal strain (LV-GLS), RV-GLS, and RV-GCS were not significantly different from controls (77). In contrast, studies on a 61-patient cohort using feature-tracking CMR (22, 24) consistently reported significantly reduced LV global radial strain (LV-GRS) and RV-GLS, with no significant differences in LV-GLS or LV-GCS. The disparity in strain parameters identified as abnormal across these studies likely reflects differences in imaging techniques (fSENC vs. feature tracking) and analysis software, rather than true biological discrepancies.

In younger populations with WD, strain parameters appear to be largely preserved. Deng et al. examined two independent young Asian cohorts (79, 110). In the first study (110), comprising 14 patients with early cardiac involvement, no significant differences were identified in RV or LV strain parameters when compared to healthy control subjects. In the second cohort (79), consisting of 41 patients, LV-GLS, LV-GCS, and LV-GRS similarly demonstrated no significant differences relative to controls, although RV strain parameters were not reported. Similarly, Johansson et al. (25, 78) examined a small cohort of 17 well-treated patients and observed a significant reduction in LV-GCS, while LV-GLS and LV-GRS remained comparable to controls. These findings suggest that strain abnormalities may be more subtle in well-treated populations and less prevalent in younger WD patients.

#### 4.3.4 Myocardial tissue characterization and mapping

Myocardial tissue characterization is a principal advantage of CMR in assessing cardiac involvement in WD. LGE imaging and multiparametric mapping enable precise identification of early myocardial damage, interstitial fibrosis, and tissue edema, with LGE serving as a classic quantitative indicator of myocardial fibrosis (12, 22, 25, 77–79, 109).

Multiple prospective CMR studies have consistently demonstrated that LGE in WD patients exhibits a characteristic distribution pattern, predominantly located at the RVIP and the mid-myocardial layer of the interventricular septum (12, 22, 24, 77, 79). Specifically, 95.1% (58/61) of WD patients exhibited pronounced LGE at the RVIP, and 18.0% (11/61) displayed mid-myocardial LGE stripes in the interventricular septum, whereas such LGE patterns were rarely observed in healthy controls (12, 22). A minority of WD patients also exhibit non-ischemic LGE in the subepicardial inferolateral, inferior, and inferolateral regions (77, 79). The extent of LGE correlates positively with UWDRS score (*r* = 0.53, *p* < 0.001) (12), suggesting an association between myocardial fibrosis and disease progression. Notably, LGE findings have been consistent across studies, supporting RVIP and mid-septal LGE as characteristic features of WD-related myocardial fibrosis, with no major discrepancies reported.

Native T1, T2 and ECV values further complement the evaluation of early myocardial involvement in WD (25, 77–79, 110). Conversely, native T2* mapping values in WD patients do not significantly differ from those observed in healthy controls (77, 109), likely due to the diamagnetic properties of copper (111). In contrast, native T1, T2, and ECV values are markedly elevated in patients with WD (77, 79, 110). A study of 14 young Asian WD patients without overt cardiac abnormalities or LGE demonstrated significantly elevated native T1, T2, and ECV (1,105.4 vs. 1,042.2 ms, 54.4 vs. 51.0 ms, and 32.7 vs. 26.5%, respectively; all *p* < 0.05) (110). A subsequent prospective study of 41 patients corroborated these findings and identified a strong positive correlation between ECV and UWDRS score (Pearson's *r* = 0.64, *p* < 0.001) (79), supporting ECV as a quantitative measure of myocardial involvement severity. Multiparametric CMR studies have also suggested coronary microvascular dysfunction, evidenced by reduced stress perfusion alongside elevated ECV (25, 78), though this derives from a single small-cohort study and requires confirmation. Overall, while LGE findings are consistent, the prognostic implications of elevated mapping parameters remain to be established.

#### 4.3.5 Clinical implications and limitations

CMR further delineates distinct phenotypic variations among different subgroups of WD patients, thereby providing substantial evidence to support risk stratification and personalized clinical management strategies (77, 79, 109, 110). In subgroup analyses, WD-neuro^+^ patients demonstrated consistently more severe myocardial injury, as evidenced by greater reductions in LV-GCS and more extensive LGE (77, 79, 109). In young WD patients (mean age <30 years), CMR enables the detection of subclinical myocardial involvement even in the absence of cardiac symptoms, preserved EF, and negative LGE, with elevated native T1, T2, and ECV (79, 110). Similarly, for asymptomatic WD patients with normal ECG and echocardiography, CMR remains highly sensitive in identifying subclinical fibrosis and subtle myocardial strain abnormalities (79, 110).

Despite its established value for myocardial tissue characterization, CMR exhibits several limitations. Its limited availability and high cost impede its use as a routine screening or longitudinal monitoring tool, especially in regions with constrained medical resources. CMR does not directly quantify myocardial copper content, and the correlation between CMR metrics (e.g., LGE, ECV) and copper deposition has yet to be validated against endomyocardial biopsy—the gold standard for copper quantification. Additionally, most WD CMR studies are limited by small sample sizes and short follow-up, limiting definitive conclusions on prognostic implications. CMR is also contraindicated in patients with severe renal insufficiency or incompatible implants. Therefore, integrating CMR with echocardiography, ECG, clinical scores (e.g., UWDRS), copper metabolism parameters, and cardiac biomarkers (e.g., troponin, NT-proBNP) is recommended for comprehensive cardiac evaluation.

Based on current evidence, CMR may be considered in patients with abnormal echocardiography, unexplained arrhythmias, elevated cardiac biomarkers, WD-neuro^+^ phenotype, young age (for baseline assessment), or prior to major therapeutic changes (e.g., liver transplantation), as summarized in Table 1. Conversely, routine CMR screening for all WD patients cannot be universally recommended due to limited availability, high cost, and lack of standardized protocols.

**Table 1:** Proposed framework for cardiac evaluation in WD: modalities, indications, key findings, and clinical implications.

Modality	Indications	Key findings in WD	Clinical implications
ECG	All WD patients at diagnosis and annually; Holter monitoring for those with cardiac symptoms or ECG abnormalities	P-wave dispersion, QT/QTc prolongation, conduction blocks, atrial/ventricular arrhythmias	Primary screening tool; normal ECG does not exclude myocardial involvement; abnormalities guide further imaging
Echocardiography	Abnormal ECG/Holter findings, cardiac symptoms, WD-neuro^+^ phenotype, or long disease duration	LV hypertrophy, reduced strain rate/segmental strain abnormalities, diastolic dysfunction, mild valvular regurgitation; concentric remodeling in pediatric patients	Detects subclinical dysfunction; advanced techniques (TDI, STE) enhance sensitivity; guides CMR referral
CMR	Abnormal echocardiography, unexplained arrhythmias, elevated cardiac biomarkers, WD-neuro^+^, young patients (may be considered for baseline assessment), or pre-liver transplant evaluation	Elevated ECV/native T1/T2, LGE at RVIP/interventricular septum, reduced strain parameters, LV clefts	Enables non-invasive tissue characterization; detects subclinical fibrosis and phenotypic variations; facilitates risk stratification and personalized management

ECG, electrocardiogram; CMR, cardiovascular magnetic resonance; WD, Wilson disease; LV, left ventricular; ECV, extracellular volume; LGE, late gadolinium enhancement; RVIP, right ventricular insertion point; TDI, tissue Doppler imaging; STE, speckle-tracking echocardiography.

In summary, this section has systematically reviewed the roles of ECG, echocardiography, and CMR in assessing cardiac involvement in WD. A comprehensive framework for cardiac evaluation—integrating these three modalities with clinical risk stratification—is provided in Table 1.

## 5 Conclusion

Cardiac involvement in WD is a clinically significant but underrecognized complication that worsens patient prognosis. This review highlights that copper-induced oxidative stress, mitochondrial dysfunction, and multiple cell death pathways (cuproptosis, ferroptosis) underlie myocardial injury, leading to a clinical spectrum ranging from subclinical abnormalities to life-threatening arrhythmias, cardiomyopathy, and SCD. Multimodal imaging—particularly CMR—provides valuable insights into early detection and risk stratification. Several critical gaps remain: the correlation between CMR metrics and myocardial copper content has not yet been validated against histopathological standards; existing cohort studies are limited by small sample sizes and heterogeneous populations; and standardized cardiac screening protocols are lacking. Future multicenter prospective studies should integrate CMR with novel biomarkers, assess the impact of early anti-copper therapy on subclinical cardiac damage, and develop evidence-based guidelines for routine cardiac evaluation in WD. Addressing these gaps will improve long-term outcomes for WD patients.
